# Changes in spontaneous overt motor execution immediately after observing others’ painful action: two pilot studies

**DOI:** 10.1007/s00221-018-5290-7

**Published:** 2018-06-07

**Authors:** Annelies Pool-Goudzwaard, Wim Groeneveld, Michel W. Coppieters, Wim Waterink

**Affiliations:** 1 Amsterdam Movement Sciences, Faculty of Behaviour and Movement Sciences, Vrije Universiteit Amsterdam, Van der Boechorststraat 7, 1081BT Amsterdam, The Netherlands; 2 Somt University of Physiotherapy, Amersfoort, The Netherlands; 3 Department of Neuroscience, Faculty of Health Sciences and Medicine, ErasmusMC University, Rotterdam, The Netherlands; 4 Menzies Health Institute Queensland, Griffith University, Gold Coast, Australia; 5 Faculty of Psychology and Educational Sciences, Open University of the Netherlands, Heerlen, The Netherlands; 6 School of Health and Rehabilitation Sciences, The University of Queensland, Brisbane, Australia

**Keywords:** Low back pain, Motor control, Action observation, Empathy, Mirror neuron system

## Abstract

Research has demonstrated that motor control is directly influenced by observation of others’ action, stimulating the mirror neuron system. In addition, there is evidence that both emotion and empathy after observing a painful stimulus affects motor cortical excitability and reaction times. Aim of the present two pilot studies is a) to test for significant influence of observing other’s painful bending of the trunk on execution of the same activity in a self-directed bending action (study 1) and to compare these results with a bending action according to a strict bending protocol (study 2). In addition to study 1, differences between Low Back Pain (LBP) patients versus healthy subjects are tested. Video footage of a (1) neutral, (2) painful, and (3) happy bending action was presented in random order. Changes in flexion–relaxation phenomenon (FRP) of back muscles were studied directly after watching the videos with surface EMG, in study 1 during a self-directed bending action in LBP patients and healthy subjects, in study 2 according to a strict bending protocol. FRP ratios were calculated by a custom-made analysis scheme tested for sufficient reliability prior to both studies. Evoked emotions were measured with an Emotional Questionnaire after each video. A Mixed Model ANOVA was used to test for the effect video and the difference between LBP and healthy subjects on the FRP-rs. Differences in evoked emotion will be tested with a Wilcoxon Signed Rank Test. In study 1, 24 healthy controls and 16 LBP patients FRP-rs were significantly influenced after observing a painful video in all subjects versus a happy and neutral video (*p* = 0.00). No differences were present between LBP and healthy controls. All subjects experienced more fear after observation of the painful video (*p* 0.05). In study 2, 6 healthy subjects followed the strict FRP bending protocol for three times after observing each video. No significant changes occurred in FRPs per video compared to FRPs of six healthy subjects carrying out the spontaneous bending activity. Observing a painful action in another person changes motor performance and increases fear in both people with and without back pain, during self-directed trunk flexion, but not during a protocolled trunk flexion.

## Introduction

Rehabilitation strategies in low back pain (LBP) patients often focus on training trunk coordination, strengthening, and endurance training of muscles of the trunk (Delitto et al. 2012). Theoretical basis for this intervention is alterations in motor control of lumbar spinal muscles demonstrated to be present in this specific patient group (D’hooge et al. 2013a, b; Dickx et al. 2008; Geisser 2007; Hodges et al. 2001, 2003; Kalichman et al. 2010; MacDonald et al. 2010; Tsao et al. 2010; Etemadi et al. 2016; Sánchez-Zuriaga et al. 2015). Typical for altered motor control is the change in paraspinal muscle activity during forward bending in these patients, the so-called flexion–relaxation phenomenon (Ahern et al. 1988; Alschuler et al. 2009; Ambroz et al. 2000; Geisser et al. 2000; Watson et al. 1997; Sanchez-Zuriaga et al. 2015). Among normal healthy subjects, activity of the lumbar paraspinal muscles during flexion initially increases, and then decreases as the ligaments begin to support the trunk as the angle of flexion increases (Alschuler et al. 2009). However, in people with LBP, this paraspinal relaxation in maximum voluntary flexion tends to be absent or decreased (Ahern et al. 1988; Alschuler et al. 2009; Ambroz et al. 2000; Colloca and Hinrichs 2005; Descarreaux et al. 2008; Geisser 2007; Maher et al. 2005; Mayer et al. 2009; Watson et al. 1997; Schinkel-Ivy et al. 2013; Sanchez-Zuriaga et al. 2015). The assumption is that this altered motor control is due to anticipation of the presence of LBP (Hodges et al. 2003; Moseley and Hodges 2005) leading to a “smudging of the brain” on the sensory motor cortex and possibly an altered neural drive of muscles (Tsao et al. 2010; Chiou et al. 2014). Motor control deficits as demonstrated by alteration of the flexion–relaxation phenomenon are indeed associated with the clinical status of people with LBP (Schinkel-Ivy et al. 2013). A significant association exists between the flexion–relaxation phenomenon ratio (FRP-r) (i.e., the ratio between activity at maximal flexion and during extension) and measures of perceived disability, a measure of clinical pain, pain-related fear as well as range of motion during flexion and elicitation of pain during straight leg raise (Alschuler et al. 2009). Based on these findings, it seems logical to train motor control deficits in LBP patients.

Since rehabilitation in people with LBP often take place in groups, especially in a multidisciplinary setting another important factor can influence motor control in these patients. Motor control of muscles is also influenced by observation of others. Observing someone performing an action is known to influence motor execution and even motor skills (Ferrari 1996; Hodges et al. 2007; Lepage et al. 2010; Vogt and Thomaschke 2007; Wulf and Mornell 2008; Murata et al. 2016; Behrendt et al. 2014; Morrison et al. 2007). The neuronal mechanism of this influence may rely on the mirror neuron system. Mirror neurons are neurons that are activated not only during the execution of an action, but also during the observation of the same action performed by someone else (Di Pellegrino et al. 1992; Fadiga and Craighero 2003; Gallese et al. 1996; Gallese 2003). Results from experimental studies demonstrate facilitation of movement execution (Villiger et al. 2011), specifically of the initiation and optimization of movement, when observing congruent action in others (Ménoret et al. 2013). Murata et al. (2016) state that control of one’s own action and the mirror neuron system are shared with the “who” system, which is related to the recognition of action.

Even more, mirror neurons are also directly influenced by emotions (Enticott et al. 2008, 2011, 2012; Gazzola et al. 2006; Budell et al. 2015). A study revealed the effect of observation of emotion in others on motor cortex excitability, providing support that direct emotion perception is closely linked to action systems (Borgomaneri et al. 2012). This is in line with several studies, indicating that empathy for people in pain may be based on ‘mirror-matching’ simulation of others’ state (Gallese 2003; Morrison 2004; Singer et al. 2004; Budell et al. 2015). Avenanti and Aglioti (2006) demonstrated that not only the affective nodes in the pain network are concerned with empathy for pain, but also the sensomotoric side (Avenanti and Aglioti 2006). Avenanti et al. (2009a, b) demonstrated that an onlooker to a needle penetration of a models hand leads to diminished cortical excitability specific for the muscle and hand observed to be penetrated. In contrast, observing a needle penetration in one other’s hand leads to a generalized corticospinal excitability of the opposite hand, leading to a possible freezing response (Avenanti et al. 2009a, b). However, the participants in these studies were not active nor any action was required. One might discuss whether motor action preparation and activity could have an effect on these results. Indeed, Morrison et al. (2007) demonstrated altered reaction times such as speeding withdrawal response and slowing approach movements with the hand/finger pressing keys when observing a needle pricking a finger (Morrison et al. 2007). Gallang et al. (2017) stated that these responses contrast muscle specific inhibition after pain observation often found in transcranial magnetic stimulation (TMS) studies. Furthermore, Gallang et al. (2017) demonstrated that participants actually responded faster (increased excitability) after observation of a painful stimulus to the hand than a non-painful stimulus, irrelevant if the participants responded with the foot or the hand. Even more, the delay (500 ms) of a Go/No go signal even increased the speed of the response. It seems that sensorimotor contagion of emotion leads to altered excitability, studied by reaction times to a Go/No go task after observing a painful stimulus. However, these studies cannot answer the question whether people with LBP in a rehabilitation group might be influenced in their overt motor activity by observing others in pain. After all, these people with LBP do not observe “a painful damaging stimulus” but a painful activity in others, nor have to respond in a predefined activity to this stimulus as quickly as possible. Still, these patients will observe others moving with pain while performing the same functional activities.

The aim of the first pilot study was (a) to demonstrate differences in an overt bending motor action measured with EMG, after watching a painful bending activity versus a bending activity in a neutral and happy condition and (b) whether this is different for people with LBP and healthy controls. The aim of the second study was to demonstrate the differences between following a strict bending protocol versus a self-directed bending action after observing the same videos.

## Materials and methods

### Design

Two fundamental experimental pilot studies have been performed. Medical ethical approval was obtained by the Open University of The Netherlands.

### Study 1

#### Participants

For the first study, healthy subjects (*n* = 24) are recruited from the Department of Neuroscience at the ErasmusMC University, Rotterdam. People with LBP (*n* = 16) are recruited from primary care physiotherapy clinic. All subjects voluntary participated in the study after reading an information flyer and signed an informed consent. Inclusion criteria were age between 20 and 60 year old en being able to read and understand Dutch. Exclusion criteria were specific LBP due to malignant processes and systematic disease as well as inability to bend forward.

#### Questionnaires

Prior to the measurements, all subjects filled in a questionnaire containing the Roland Disability Questionnaire (RDQ), a pain Numeric Rating Scale (NRS) and additional questions on socio-demographic data. The Roland disability questionnaire has proven sufficient to good reliability and validity to measure disability due to LBP (Smeets et al. 2011). The NRS is a scale between 0 (no pain) and 10 (excruciating pain). The psychometric qualities of both instruments are good (Ostelo and de Vet 2005; Soer et al. 2012; van der Roer et al. 2006). With a positive score on the RDQ > 0 and NRS > 0, a subject entered the LBP group. Prior and during the measurements, an “Emotional Questionnaire” was used. This is a small questionnaire scoring six separate emotions (surprise, happiness, fear, irritation, disgust, and sadness) on a five-point Likert scale from not at all present (0) to very strong (5).

#### Bending motor action–flexion relaxation phenomenon

In the literature, the flexion–relaxation phenomenon (FRP) is described as relaxation of the paraspinal Erector Spinae muscle in full flexion. This can be measured by Surface Electro-MyoGraphy (SEMG) during a predefined protocol of a few seconds bending forward, a few seconds maximal flexion and a few seconds extension of the lumbar spine (Ahern et al. 1988; Alschuler et al. 2009; Ambroz et al. 2000; Geisser et al. 2000; McGorry and Lin 2012; Watson et al. 1997). We hypothesized that after watching a painful bending action, the motor excitability of the trunk muscles would be increased and that the relaxation of the erector spinae muscles would not occur while bending, even more evident in LBP patients.

**Fig. 1:**
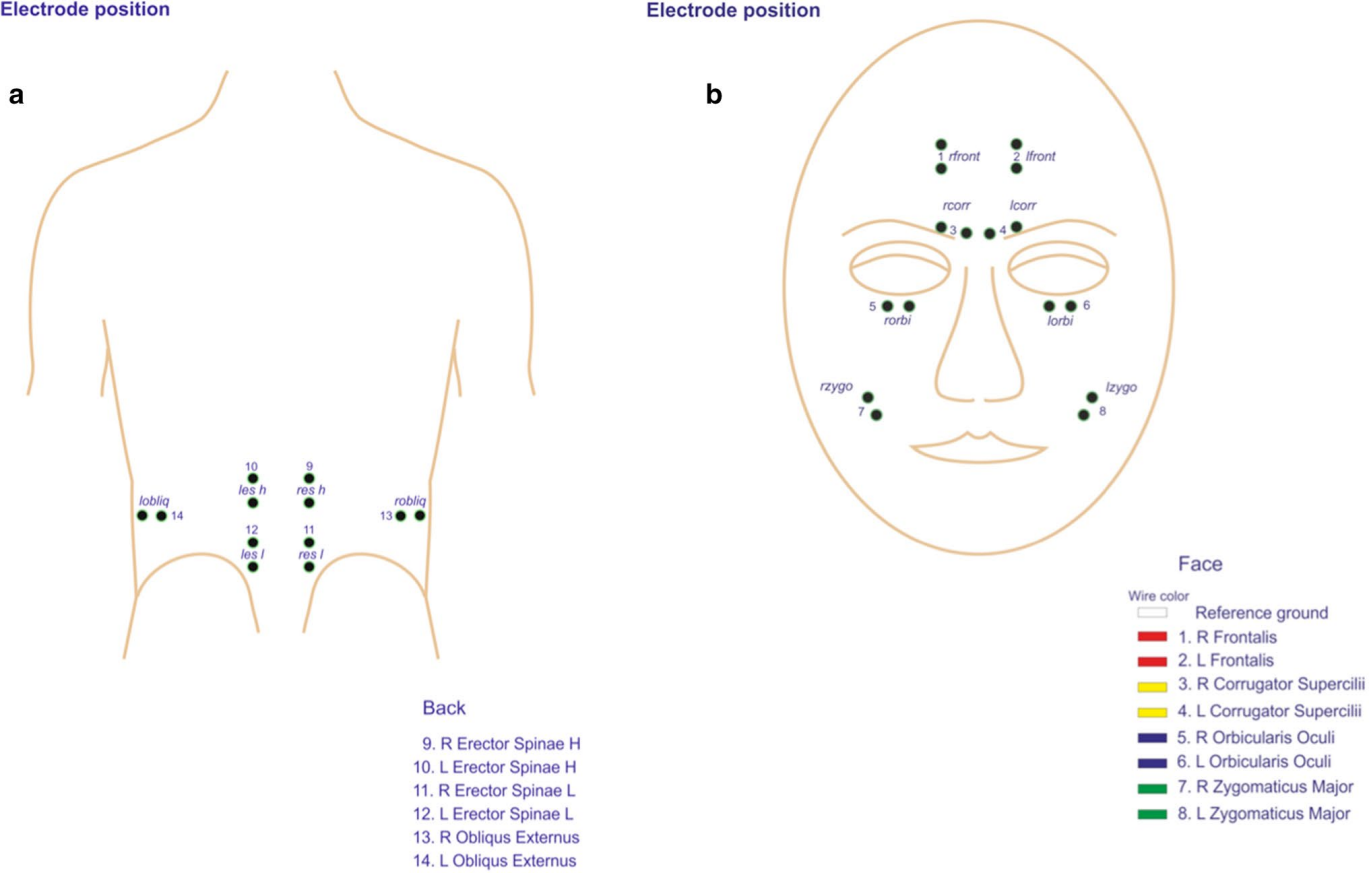
Electrode position of the back muscles (**a**) and on the face (**b**)

For the first experiment, we decided that the official flexion relaxation protocol being a predefined time framed protocol of flexion and extension of the trunk did not reflect normal movement patterns as bending forwards in healthy subjects nor in LBP patients during rehabilitation programs. We decided to focus on a spontaneous change in the flexion relaxation phenomenon evoked by observing a similar bending activity in others. Therefore, we adapted the flexion relaxation protocol to a more spontaneous activity as picking up an object, like a wallet from the ground. At first, we established a mean standardized time frame by measuring the speed of picking up a wallet from the ground multiple times prior to the research. A mean standardized time frame could be calculated of 0.1-s maximal voluntary flexion after bending forward picking up the wallet and 0.3 s coming up straight. During the experiment, continuous SEMG measurements were performed of the erector spinae muscles high (height L1) and low (height L4) (see Fig. 1a). All EMG electrodes were connected to a portable EMG registration system (TMS porti: Twente Medical Systems International, Oldenzaal, The Netherlands). EMG signals were band pass filtered (10–1000 Hz). A Notch filter of 50.2–49.8 Hz suppressed possible power-line interference of 50 Hz.

For study 1, we measured not only erector spinae activity with SEMG, but also facial muscles: the frontalis, corrugator supercilii, orbicularis oculi, and zygomaticus major on the left and right sides (Lapatki et al. 2010) (see Fig. 1b).

**Fig. 2:**
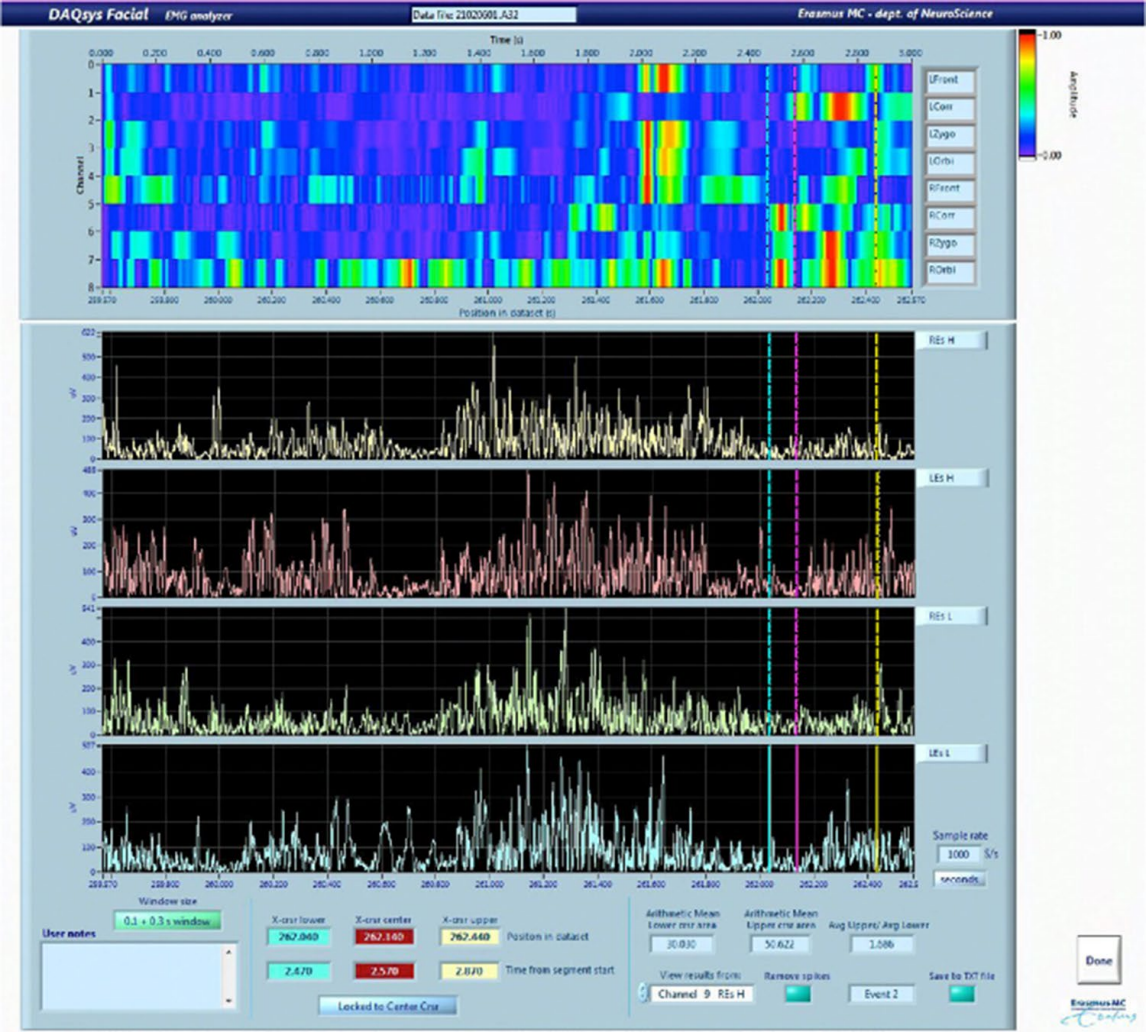
Three seconds of SEMG data registered after the marker signalling picking up a wallet. The upper part shows the intensity graph of facial muscles, the lower part shows the 4 channels of the erector spinae muscles (9, left erector spinae high; 10, right erector spinae high; 11, left erector spinae low; 12, right erector spinae low). The 0.1 s of maximal flexion and 0.3 s of extension as a fixed time frame are selected at the location of most increased facial activity

Increased activity in facial muscles can be used in two ways. At first, increased activity of facial muscles can be used as a reference for maximal bending forward, since activity in facial muscles increases during extension after bending forward. An intensity graph of SEMG of the facial muscles plotting the amplitude in color versus time demonstrated the highest activity in facial muscles related to bending forward and maximal flexion (see Fig. 2). SEMG data during the mean standardized time frame of 0.1 s of maximal flexion and 0.3 s extension with the highest facial activity were used for analysis. Second, increased activity of facial muscles can reveal the subjects’ affective state evoked by the videos (de Wied et al. 2006; Ekman et al. 1981; Lapatki et al. 2010; Larsen et al. 2003). These positive and negative affective states can be reliably distinguished by facial EMG (Larsen et al. 2003).

#### Experimental setup and protocol

**Fig. 3:**
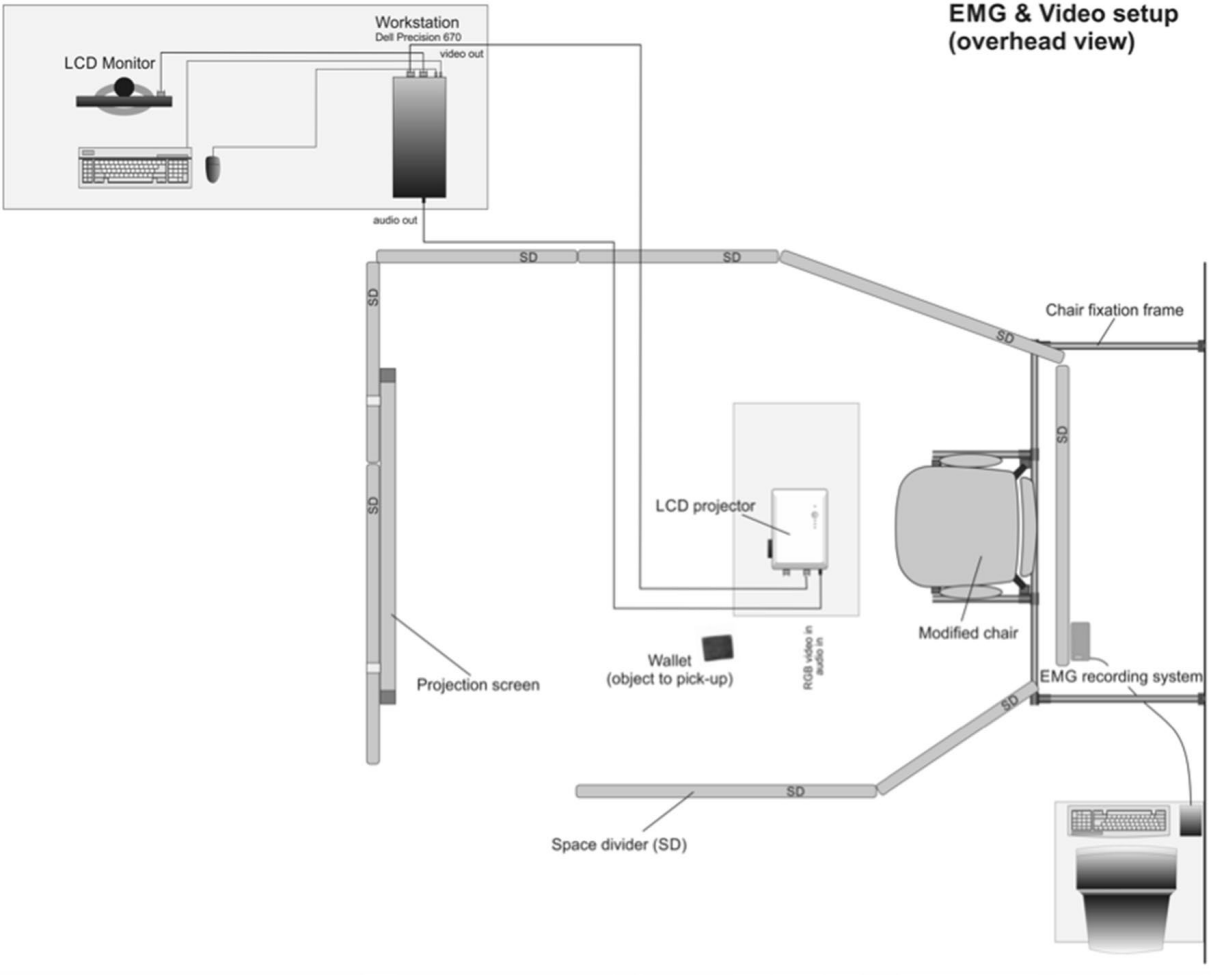
Experimental setup

All subjects were measured in a separate area, created by room dividers, to avoid emotional contagion and EMG activity by the presence of the researchers (see Fig. 3). All subjects sat on a chair behind a table on which two printed emotional questionnaires were placed. All subjects watched three separate custom-made videos: (a) a video with a person picking up a wallet from the floor extending to erect posture and walking on (neutral video); (b) picking up the same wallet hardly able to raise himself to erect posture due to excruciating acute LBP (painful video); and (c) picking up the same wallet coming to erect posture with someone else responding enthusiastic and glad that their wallet was found (happy video). The videos were displayed in random order on a projection screen in front of them, one ‘condition’ video (3 conditions neutral, happy and painful) per subject. We choose not to repeat any videos to mimic ‘normal life observing other subjects bending and raising again as well as LBP patients in a rehabilitation clinic since viewing another LBP patient hurting his or her back, hardly able to raise again. It is not natural for such a patient to repeat this movement over and over. This indicated that we could only execute one bending action per video. Prior to study 1, we tested the test–retest reliability in ten healthy subjects executing 3 × 3 bending actions (ICC 0.67 during flexion and 0.78 during extension). Since ICC was sufficient, we decided that one measurement could be used per video in study 1. During study 1, the bending action was carried out by placing a wallet on a mark on the ground beside the table. After each video, the subject was instructed to pick up the wallet and to sit down on the chair and fill in one emotional questionnaire. After completion, the subject was instructed to return the wallet to the marking on the ground. All separate actions (start video, emotion evoking moment during the video, ending of the video, standing up, bending forward, return to chair, etc.) were marked with a marker on the 16th channel of the SEMG-recording system. After the final video, the subjects were asked to stand erect for 3 s, while three consecutive SEMG “rest activity” measurements were performed of the erector spinae muscles to make it possible to normalize SEMG data by dividing the SEMG value during the flexion and extension by the mean average SEMG during standing of the same erector spinae muscle.

#### Data analysis

**Fig. 4:**
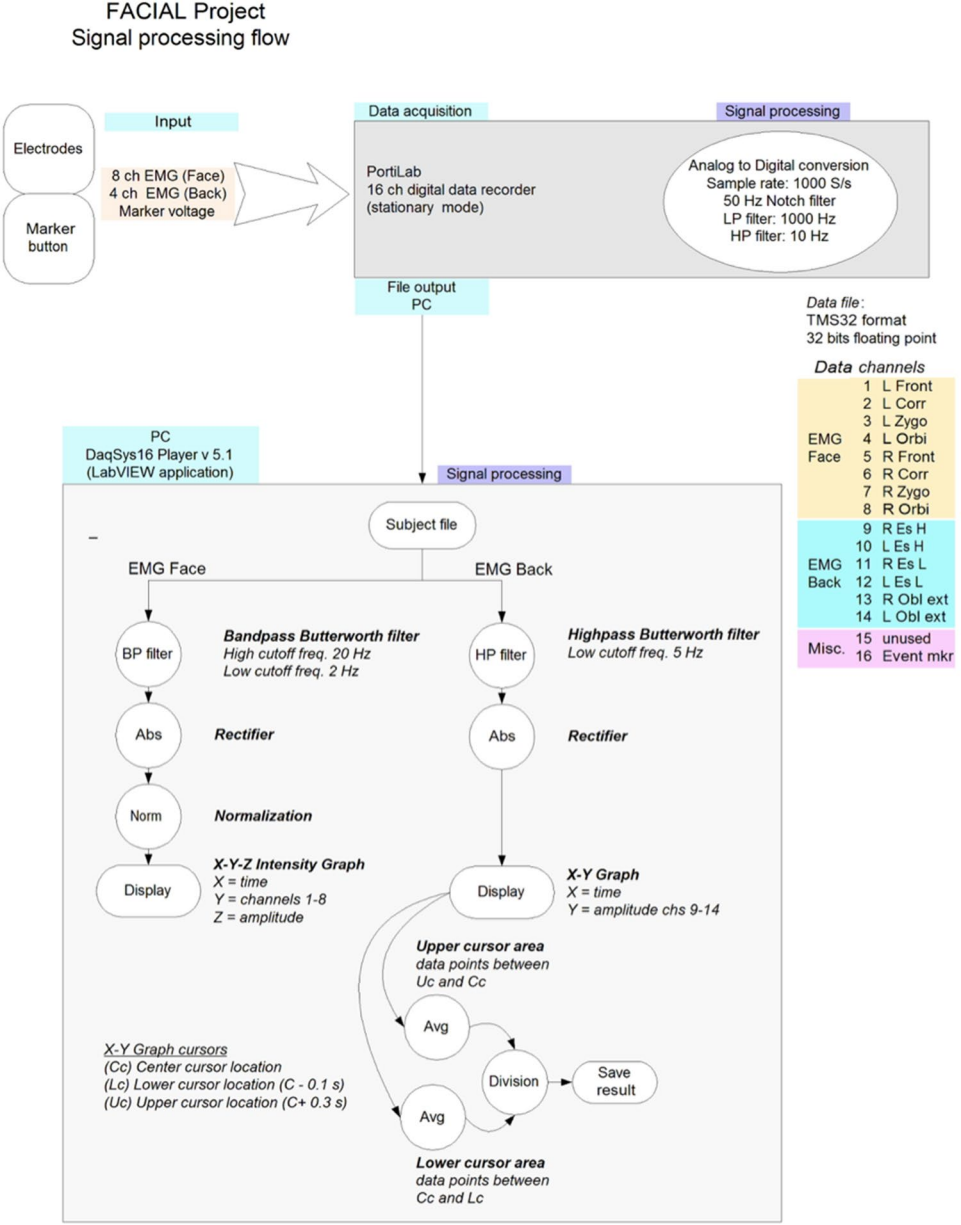
Signal flow and processing

In study 1, data analysis was performed in a custom-made LabVIEW^®^ 8.2 application-DaqSys 16 player. Three complete seconds of data after markers 3 and 9 (indicating picking up the wallet from the floor directly after watching the videos) were used. The FRP was localized by studying SEMG activity of the erector spinae muscles (four channels) (see Fig. 1). At first, SEMG data during the flexion relaxation phenomenon of all erector spinae channels were normalized by dividing the maximum SEMG during extension and the average SEMG during maximal flexion by the mean SEMG of 3 s of rest activity in standing. According to the strict analyses protocol to define the Flexion–Relaxation Phenomenon ratio (FRP-r) with the highest association with clinical measures in LBP patients as pain and disability (Alschuler et al. 2009), the FRP-r was calculated by dividing the normalized maximum SEMG during extension (0.3 s) to the average normalized SEMG during maximal voluntary flexion (0.1 s) (Alschuler et al. 2009), see Fig. 4 for the signal processing flow of the SEMG. Reproducibility of this procedure in observation of a pain-free video was tested with 3 × 3 repetitions per video in ten healthy subjects, prior to this research and demonstrated an intra-class correlation coefficient of 0.67 when calculating normalized values for maximal voluntary flexion and 0.78 for normalized extension.

Affective facial EMG responses were calculated using the smoothed, rectified maximum EMG output during a short interval of 100 ms after each marker related to the emotion evoking moment in the pain and happy videos. These max. EMG values per muscles and side were standardized as a proportion of 3 s of baseline EMG amplitudes per subject (Boxtel van 2010). Higher proportions of zygomaticus major and orbicularis oculi muscle activity are expected during an elementary positive emotion of happiness, while frontalis and corrugator supercilii muscle activity is expected during a pain-related or negative emotion like fear, anger, or sadness (Boxtel van 2010).

#### Statistical analysis

To demonstrate changes in emotional status after observation of a painful, happy, or neutral video, at first, all scores were calculated for change from the initial values on the emotional questionnaire to eliminate confounding effect of higher scores on emotional status prior to the measurement. These delta scores per video were tested for significant differences overall and per group (LBP versus healthy subjects) using a Wilcoxon signed rank test and a Mann–Whitney *U* test, respectively.

Differences in affective state (positive versus negative facial EMG muscle activity) evoked by the videos were tested for significant difference between positive and negative facial mimicry using a Paired *T* test.

A Mixed Model ANOVA was used to test for the effect of the observed video and the difference between LBP and healthy subjects on the FRP-rs, with the three video conditions (Painful, Happy, and Neutral) as a within-subjects factor and participant type (LBP and Healthy) as the between-subjects factor. Possible confounding effect of order of videos was taken into account. All data were tested for normal distribution. Logistic transformation of all skewed data was carried out prior to statistical analysis.

The same Mixed Model ANOVA was used to test for the effect in the second study.

### Study 2

#### Subjects

For the second study, students and employees of the VU University in Amsterdam were asked to participate. With a difference in FRP-rs of 1.1 between the healthy subjects watching a painful (2.6 ± 0.9) versus a neutral video (1.55 ± 0.7) and the expectation that this similar difference will be present between a flexion relaxation phenomenon according to a strict protocol and a non-strict protocol with an alpha of 0.05 and a power of 0.8, we need a sample size of *n* = 12 (*n* = 6 for the strict protocol and *n* = 6 for the spontaneous action). The last group will be derived from study 1 by an ad random selection of every fourth healthy subject. Another six healthy subjects participated in study 2.

#### Protocol

The protocol described by McGorry and Lin (2012) was followed to measure the FRP in study 2 with standing still for 4 s, bending forwards in 4 s, remaining fully flexed for 4 s and extending for 4 s. For study 2, we recorded and calculated the movements of the spine using an optical tracking system Optotrak, reading two markers on the dorsal side of the spine at the height of L1 and L5. A marker signal was used to synchronize the data of the EMG with the output of Optotrak.

Data analyses were performed in a custom-made Matlab^®^ R2014b application. Again, all SEMG data during the flexion relaxation phenomenon of all erector spinae channels were normalized. During four seconds of maximal flexion measured by Optotrak, mean SEMFG of a same time frame as in study 1 of 0.1 s was calculated as well as 0.3 s during the extension. According to the same pre-described protocol, FRP-rs were calculated as in study 1.

#### Statistical analyses

Another Mixed Model ANOVA was used to test for the effect of the observed video with the three video conditions (Painful, Happy, and Neutral) as a within-subjects factor. As between-subjects factor the effect of either or not following, a strict FRP protocol was used.

## Results

As described, 40 subjects participated in study 1 16 LBP patients and 24 healthy controls and 6 healthy subjects in study 2. Socio-demographic data are shown in Table 1. No significant differences are present between LBP patients and healthy controls on socio-demographic data and initial values on the EQ. Cronbach’s alpha for internal consistency for positive labeled emotions on the EQ (surprise and happiness) was 0.67. The Cronbach’s alpha of the negative labeled emotions (fear, irritation, disgust, and sadness) was 0.88.

**Table 1:** Socio-demographic data and initial values on the EQ

Subjects	All (*n* = 40)	HC (*n* = 24)	LBP patients (*n* = 16)	HC study 2
Gender (F/M)	19/21	11/13	8/8	4/2
Age (years)	35 (SD ± 12.1)	34 (SD ± 12.3)	37 (SD ± 11.9)	27 (SD ± 11.9)
Outcome questionnaires				
NRS (mean SD, min, max)		0	4 (SD ± 2.5) min 2, max 7	0
RDQ		0	5.2 (SD ± 4, min 0, max 19)	0
EQ				
Surprise		1 (SD ± 0.6)	1 (SD ± 1.1)	0 (SD ± 0.3)
Happiness		2 (SD ± 0.7)	1.5 (SD ± 1.0)	0 (SD ± 0.6)
Fear		0 (SD ± 0.2)	0 (SD ± 0.4)	0 (SD ± 0.1)
Irritation		0 (SD ± 0.2)	0 (SD ± 0.7)	0 (SD ± 0.2)
Disgust		0 (SD ± 0.1)	0 (SD ± 0.3)	0 (SD ± 0.1)
Sadness		0 (SD ± 0.3)	0 (SD ± 0.5)	0 (SD ± 0.1)

*LBP* low back pain, *SD* standard deviation, *F/M* female/male, *NRS* numeric rating scale, *RDQ* Roland disability questionnaire, *EQ* emotional questionnaire

### Evoked emotion

All median scores on changes in emotional status after observation of the videos are 0 (see Table 2 for study 1). In the overall group, there is a significant difference of feeling more fear (*p* < 0.05) after observation of a painful action in both groups with respect to the neutral and happy video in study 1. This, however, is not present in study 2. In study 1, significant less fear is present in healthy controls after watching a happy video with respect to the pain patients.

**Table 2:** Mean changes in scores per emotion on the emotional questionnaire after observation of a painful (P), neutral (N) and a happy (H) video, overall and per subgroup in study 1

Subjects	All (*n* = 40)			HC (*n* = 24)			LBP patients (*n* = 16)		
EQ (± SD)	N	P	H	N	P	H	N	P	H
Surprise	0.33 (0.7)	0.23 (0.7)	− 0.03 (0.9)	0.30 (0.7)	0.22 (0.6)	0.0 (0.9)	0.38 (0.7)	0.25 (0.9)	− 0.8 (0.8)
Happiness	0.08 (0.6)	− 0.03 (0.7)	− 0.67 (0.7)	− 0.04 (0.5)	− 0.13 (0.7)	0.65 (0.7)	0.25 (0.7)	0.13 (0.6)	0.69 (0.6)
Fear	0.05 (0.2)	− 0.08* (0.3)	0.03 (2.9)	0.4 (0.2)	0 (0)	− 0.4* (0.2)	0.06 (0.2)	− 0.06 (0.4)	0.15 (0.3)
Irritation	0.03 (0.2)	0.08 (0.4)	0.22 (0.6)	0.5 (0.2)	0.13 (0.5)	0.13 (0.5)	0 (0)	0 (0.4)	0.38 (0.9)
Disgust	0 (0)	0,13 (0.5)	0 (0.2)	0 (0)	0.14 (0.4)	0 (0)	0 (0)	0.13 (0.6)	0 (0.4)

No significant differences are present neither between both videos nor between patients versus healthy subjects

*p < 0.05

In contrast with our expectation, EMG facial activity in the 100 ms interval after the marker related to emotion evoking moment did not show a single peak of muscle activity after a “still” period. Multiple peaks in muscle activity were present throughout all videos in all facial muscles. Since low-frequency artifacts such as eye blinking and/or other movements like activity of neighboring muscles, swallowing, etcetera were interfering with the facial data we questioned these data for reliability and did not perform statistical analysis on this data.

### Influence of observation of pain on FRP-rs

**Graph 1:**
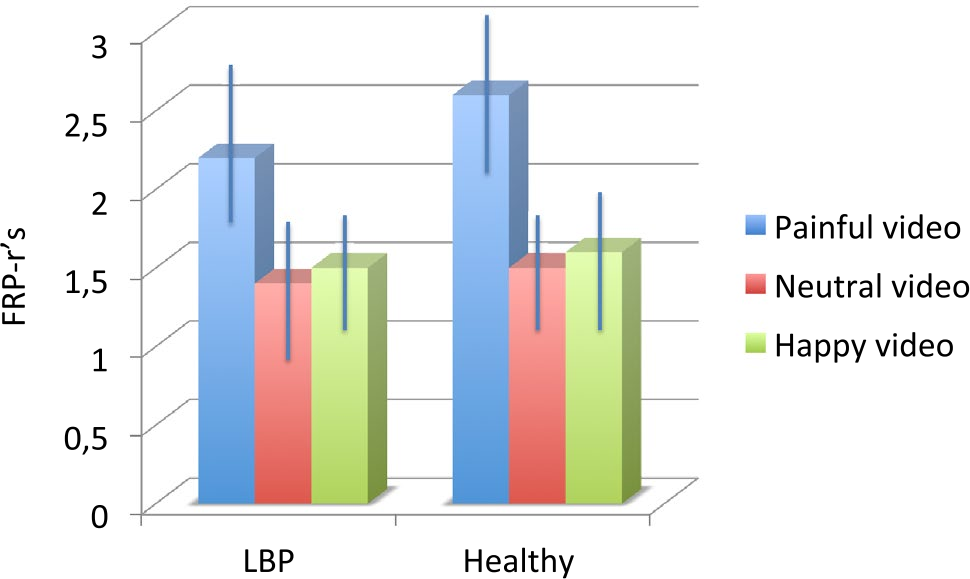
Mean FRP-rs ± standard deviation (SD) per video in LBP patients and healthy controls

Differences between FRP-rs per video are displayed in Graph 1. Higher FRP-rs are present in the painful condition versus the neutral and happy condition, indicating relatively more flexion relaxation (less activation) of the erector spinae at full trunk flexion.

Since sphericity was not present (*χ*^2^ = 1.2, *p* > 0.05, *df* 1.98 was corrected by Greenhouse–Geisser. The mixed model ANOVA demonstrates a significant effect of video as a within subject effect *F* = 3.26, *p* < 0.05. Follow-up paired *t* tests demonstrated a significant difference in (log transformed) FRP-rs after observing the painful video (Mn 0.72, SD 0.37) and after observing the neutral video {Mn 0.23, SD 0.52 [*t*(60) = 6.83 *p* = 0.00]} as well as the happy video [Mn 0.31, SD 0.58, *t*(60) = 4.87, *p* = 0.00]. No significant differences were present in FRP-rs after observing a happy or a neutral video. The between-subjects effects were not significant. No significant effect was present by order of video.

**Graph 2:**
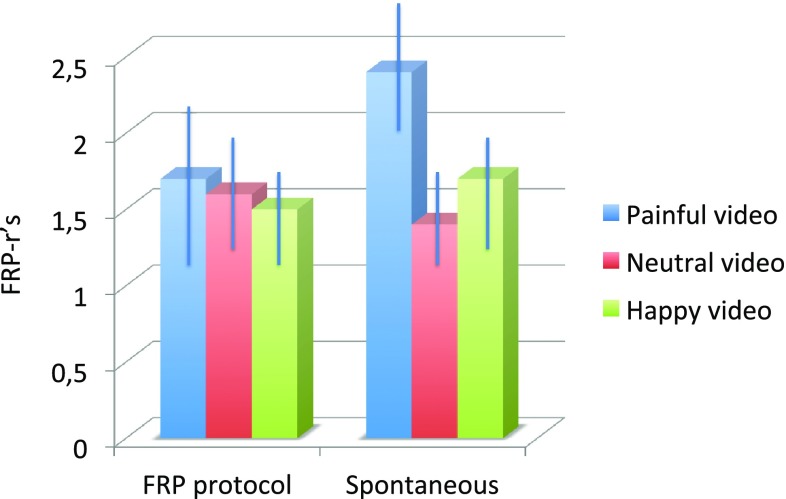
Mean FRP-rs/standard deviation (SD) per video in healthy subjects with a strict FRP protocol versus spontaneous bending action

No significant within-subjects effects were present of observing a video nor between-subjects effects in the strict protocol condition between in study 2 (see Graph 2).

## Discussion

Main finding of the current pilot study is that observing an overt painful motor bending action of the trunk increases fear in the observers and significantly alters the execution of the congruent painful action. It seems, this only takes place when the motor activity is spontaneous and immediately after observing other’s painful action. The fact that observing others’ painful motor action alters one’s own motor action is relevant, especially for LBP patients training together always observing others’ painful action. The higher FRP-rs present while observing a painful bending action, in all subjects, indicate relatively more flexion relaxation (less activation) of the erector spinae at full trunk flexion. This is not present after observing a neutral or happy bending condition. From a clinical point of view it is relevant that in all subjects observation of pain alters motor execution of a spontaneous similar action. LBP patients often display pain behavior when bending forward or lifting objects. Meanwhile, LBP patients, especially with a chronic condition, are often trained in groups with an emphasis on graded activity. Seeing another patients´ pain behavior might interfere with a patients’ own motor control. Clinicians should be aware of this phenomenon. In addition, execution of motor control of healthy spouses or friends of these patients might be influenced as well. Still, we have to be careful in drawing firm conclusions on both pilot studies. Confirmation of these findings in a larger study is necessary for more robust proof.

Altered FRP-rs are a new finding which is supplementary to the knowledge we have on the influence of observation others’ pain on motor excitability. It seems that not only motor excitability and reaction times in motor execution alters after observation of a painful stimulus to the hand, but also by seeing others’ painful activity executing the same action (Ferrari 1996; Hodges et al. 2007; Lepage et al. 2010; Vogt and Thomaschke 2007; Wulf and Mornell 2008; Murata et al. 2016; Behrendt et al. 2014; Morrison et al. 2007).

### Emotional status

The current study has demonstrated that the emotional status of the observer can significantly be influenced directly by a negative stimulus like observing others’ pain. All subjects scored more fear on the emotional questionnaire after observing a painful video. The findings are in line with results from the literature. Data have been presented on the influence of observation of pain in humans. Seeing painful or unpleasant stimuli may elicit arousal or aversion (personal distress) reactions (Williams 2002). We have to be careful to draw firm conclusions, since the reliability of the emotional questionnaire is not tested and the data cannot be confirmed by facial mimicry (EMG of facial muscles). Furthermore, the number of subjects is small.

### Influence of observation of others’ pain on motor execution in all subjects

It has been reported in the literature that the intensity of someone else’s pain is negatively correlated to changes in cortical excitability of the observer, in healthy subjects (Avenanti and Aglioti 2006; Singer et al. 2004). Furthermore, it has been reported that negative stimuli prior to the observation of transitive hand movements facilitated cortical spinal excitability as well (Enticott et al. 2012; Hill et al. 2013). In addition, Hajcak et al. (2007) have been demonstrating increased motor cortex excitability in participants who viewed pleasant and unpleasant compared to neutral images. Although we did not study motor cortex excitability but motor execution, we could not demonstrated influence of a pleasant (happy) stimulus. Our findings do seem to support the other above-mentioned studies by demonstrating significant differences in motor execution by observing others’ pain. This is in line with the finding of motor response changes to observing others’ pain in a pain population (amputees) (Fitzgibbon et al. 2010a, b). Since motor execution changed in all subjects after observing a painful video and this phenomenon did not occur after observing a happy or neutral video, it seems that especially, the negative stimulus of observing others’ pain was the trigger. However, from the current study, we cannot conclude which processes take place within the brain. The overall emotional status of more fear in all subjects and/or empathy for pain and/ or ‘mirror-matching’ simulation of others’ state (Gallese 2003; Morrison 2004; Singer et al. 2004) or even other pain processes can play a role. The fact that in the healthy subjects in study 2, no significant change in evoked emotion was present and that also no effect was present in change in motor execution could indicate that emotions can play a role. Further research is necessary to demonstrate which pain processing take place during observation of someone else’s pain and execution of motor control.

### No differences between healthy subjects and low back pain patients

We anticipated for differences to occur after observing others’ pain between LBP patients and healthy subjects both in evoked emotion as in motor execution. However, no significant differences were present. Valeriani et al. (2008) suggested that being in pain might bias the empathic relation with others (Valeriani et al. 2008). Since no differences at group level are present in emotional status after observing others’ pain, we cannot confirm this suggestion.

Furthermore, no significant differences were present in execution of motor control during flexion of the spine, since in all subjects, the FRP-rs altered significantly. Despite the fact that research demonstrated that recalled pain as in physical pain conditions reveals significant activation in mostly similar affective pain processing brain structures, including bilateral anterior insula, anterior cingulate cortex (ACC), and thalamus (Fairhurst et al. 2012), this does not lead to different executions of motor control in LBP patients. Actually, to what extend recall of pain can play a role in motor execution is not clear. Further research is necessary to study a possible influence of the pain resonance system in pain patients on motor execution.

### Strengths and limitations

Strength of the current study is that this is the first study focusing on motor execution in relation with the model of empathy observing others’ low back pain.

Main limitation of the study is that only one trial per video has been performed in study 1. Therefore, we tried to test the effect of repeated measurements on motor execution prior to study 1. Calculating a mean of three trials would have been more robust. However, the effect of observing a video on spontaneous motor execution was not present during repeated measures according to FRP protocol.

Regarding the spontaneous motor execution, the fact that the test–retest reliability in healthy subjects after observing a neutral video was sufficient supports the reliability of the data despite the fact that only one trial per video per subject was performed. Still, the findings are not very robust. This is why we recommend a repetition of the study in a larger sample size.

In the current study, we have chosen to measure the FRP-r during a normal activity like picking up a wallet. Reason for this was the fact that a video of a flexion of the lumbar spine during a preprogrammed time schedule would interfere with the execution of the same spontaneous action as in the video. Furthermore, research does underpin the notion that mirror neurons are more sensitive to “object- and goal-orientated” movement (Enticott et al. 2011). Therefore, picking up a wallet is more appropriate in stimulating mirror neurons, but is different from the preprogrammed timed flexion–relaxation phenomenon described in the literature, which was carried out in study 2 (Ahern et al. 1988; Alschuler et al. 2009; Ambroz et al. 2000; Geisser 2007; McGorry and Lin 2012; Watson et al. 1997). Indeed, study 2 did not result in any positive findings. The scores of study 1 are not comparable to the outcome on FRP-rs in the literature. Although we have chosen the most optimal analysis of FRP-r with regard to association with clinical outcomes in LBP patients (Alschuler et al. 2009; McGorry and Lin 2012), we wonder whether these associations also count for the FRP-rs described in the current study. Still, the current data analysis of FRP-rs in normal spontaneous action seems promising and is reliable in the test–retest. Recommendation is made to test the spontaneous FRP for differences in LBP patients and healthy subjects in larger groups and to test for associations with clinical outcomes in future research.

Another limitation of the study was that validation of emotion by facial mimicry during observation of each video was not possible due to the artifacts in the facial muscles EMG recordings. It might be that crosstalk is present between the EMG electrodes: the phenomenon that electrical activity generated by a specific muscle spreads to adjacent areas through volume conduction (Boxtel 2001). Another impeding factor might have been that emotional experiences under natural circumstances often consist of a mixture of elementary emotions, which in addition, may rapidly change so that EMG response patterns may thus be a function of such undetermined or dynamic emotional states (Boxtel van 2010). Furthermore, the human face does not only display affective responses, but also produces a large variety of activities unrelated to emotional processes like speech, mental effort or mental fatigue, task involvement, startle reflexes, etc. (Boxtel van 2010). Hence, it was not possible to test the Emotional Questionnaire thoroughly for its external validity. Additional research is necessary to validate the questionnaire.

Limitation of the current study is the small sample size. Further research in larger cohorts is necessary to confirm the findings of the current study. Furthermore, the data might be disturbed by selection bias. All healthy subjects were volunteers from the department of Neuroscience. Yet, none of them knew the research questions and hypothesis, so the influence on selection bias is not large.

Another limitation of this study was that per condition (Happy, Neutral, Painful) only one video was viewed to all subjects. This is in contrast with (Avenanti and Aglioti 2006; Avenantie et al. 2009; Gallese 2000), Morrison et al. (2007), and Galang et al. (2017) who repeatedly > 18 times showed the painful stimulus video. In our view, since our experiment tends to relate the findings to the clinic, we could not show the painful video over and over again. After all, it is unusual in the clinic when an LBP patient is hurting his or her back during bending and is hardly able to rise anymore that he or her will repeat this action. We acknowledge by only demonstrating the video once we introduce a higher change on accidental findings.

Another limitation to our study is the unbalanced sample size in study 1. We were hampered in the inclusion of healthy subjects, possibly due to longer duration of data collection. After a long period of no inclusion despite several actions to increase our member, we decided to settle for a convenient sample, however, unbalanced.

## Conclusion

Observing others’ painful action increases fear and can alter spontaneous motor control during execution of the same activity in LBP patients as well as in healthy subjects. This only occurs immediately after observing the painful video and is not present during strict protocolled movements.

## References

[CR1] Ahern DK, Follick MJ, Council JR, Laser-Wolston N, Litchman H (1988). Comparison of lumbar paravertebral EMG patterns in chronic low back pain patients and non-patient controls. Pain.

[CR2] Alschuler KN, Neblett R, Wiggert E, Haig AJ, Geisser ME (2009). Flexion-relaxation and clinical features associated with chronic low back pain: A comparison of different methods of quantifying flexion-relaxation. Clin J Pain.

[CR3] Ambroz C, Scott A, Ambroz A, Talbott EO (2000). Chronic low back pain assessment using surface electromyography. J Occup Environ Med.

[CR4] Avenanti A, Aglioti SM (2006). The sensorimotor side of empathy for pain. Psychoanalysis and neuroscience.

[CR5] Avenanti A, Minio-Paluello I, Bufalari I, Aglioti S (2009). The pain of a model in the personality of an onlooker: Influence of state-reactivity and personality traits on embodied empathy for pain. NueroImage.

[CR6] Avenanti A, Minio-Paluello I, Sforza A, Aglioti S (2009). Freezing or escaping? Opposite modulations of empathic reactivity to the pain of others. Cortex.

[CR7] Behrendt F, de Lussanet MH, Wagner H (2014). Observing a movement correction during walking affects evoked responses but not unperturbed walking. PLoS One.

[CR8] Borgomaneri S, Gazzola V, Avenanti A (2012). Motor mapping of implied actions during perception of emotional body language. Brain Stim.

[CR9] Boxtel Av (2001). Optimal signal bandwidth for the recording of surface EMG activity of facial, jaw, oral, and neck muscles. Psychophysiology.

[CR10] Boxtel van A (2010) Facial EMG as a tool for inferring affective states. In: Paper presented at the Proceedings of Measuring Behavior, 2010. pp 104–108

[CR11] Budell L, Kunz M, Jackson PL, Rainville P (2015). Mirroring pain in the brain: emotional expression versus motor imitation. PLoS One.

[CR12] Chiou SY, Shih YF, Chou LW, McGregor AH, Strutton PH (2014). Impaired neural drive in patients with low back pain. Eur J Pain.

[CR13] Colloca CJ, Hinrichs RN (2005). The biomechanical and clinical significance of the lumbar erector spinae flexion-relaxation phenomenon: a review of literature. J Manipulative Physiol Ther.

[CR14] Delitto A, George SZ, van Dillen L, Whitman JM, Sowa GM, Shekelle P, Denninger TK, Godges JJ (2012). Low back pain; Clinical practice guidelines linked to the international classification of functioning, disability and health from the orthopaedic section of the american physical therapy association. J Orthop Sports Phys Ther.

[CR15] D’hooge R, Hodges P, Tsao H, Hall L, MacDonald D, Danneels L (2013). Altered trunk muscle coordination during rapid trunk flexion in people in remission of recurrent low back pain. J Electromyogr Kinesiol.

[CR16] D’hooge R, Cagnie B, Crombez G, Vanderstraeten G, Achten E, Danneels L (2013). Lumbar muscle dysfunction during remission of unilateral recurrent nonspecific low-back pain: evaluation with muscle functional MRI. Clin J Pain.

[CR17] de Wied M, van Boxtel A, Zaalberg R, Goudena PP, Matthys W (2006). Facial EMG responses to dynamic emotional facial expressions in boys with disruptive behavior disorders. J Psychiatr Res.

[CR18] Descarreaux M, Lafond D, Jeffrey-Gauthier R, Centomo H, Cantin V (2008). Changes in the flexion relaxation response induced by lumbar muscle fatigue. BMC Musculoskelet Disord.

[CR19] Di Pellegrino G, Fadiga L, Fogassi L, Gallese V, Rizzolatti G (1992). Understanding motor events: a neurophysiological study. Exp Brain Res.

[CR20] Dickx N, Cagnie B, Achten E, Vandemaele P, Parlevliet T, Danneels L (2008). Changes in lumbar muscle activity because of induced muscle pain evaluated by muscle functional magnetic resonance imaging. Spine.

[CR21] Ekman P, Hager JC, Friesen WV (1981). The symmetry of emotional and deliberate facial actions. Psychophysiology.

[CR22] Enticott PG, Johnston PJ, Herring SE, Hoy KE, Fitzgerald PB (2008). Mirror neuron activation is associated with facial emotion processing. Neuropsychologia.

[CR23] Enticott PG, Kennedy HA, Bradshaw JL, Rinehart NJ, Fitzgerald PB (2011). Motor corticospinal excitability during the observation of interactive hand gestures. Brain Res Bull.

[CR24] Enticott PG, Harrison BA, Arnold SL, Nibaldi K, Segrave RA, Fitzgibbon BM (2012). Emotional valence modulates putative mirror neuron activity. Neurosci Lett.

[CR25] Etemadi Y, Salavati M, Arab AM, Ghanavati T (2016). Balance recovery reactions in individuals with recurrent nonspecific low back pain: effect of attention. Gait Post.

[CR26] Fadiga L, Craighero L (2003). New insights on sensorimotor integration: from hand action to speech perception. Brain Cogn.

[CR27] Fairhurst M, Fairhurst K, Berna C, Tracey I (2012). An fMRI study exploring the overlap and differences between neural representations of physical and recalled pain. PloS One.

[CR28] Ferrari M (1996). Observing the observer: Self-regulation in the observational learning of motor skills. Dev Rev.

[CR29] Fitzgibbon BM, Enticott PG, Rich AN, Giummarra MJ, Georgiou-Karistianis N, Tsao JW (2010). High incidence of ‘synaesthesia for pain’in amputees. Neuropsychologia.

[CR30] Fitzgibbon BM, Giummarra MJ, Georgiou-Karistianis N, Enticott PG, Bradshaw JL (2010). Shared pain: from empathy to synaesthesia. Neurosci Biobehav Rev.

[CR31] Gallang C, Naish K, Arbabi K, Obhi S (2017). Observing painful events in others leads to a temporally general response facilitation in the self. Exp Brain Res.

[CR32] Gallese V (2000). The inner sense of action. agency and motor representations. J Conscious Stud.

[CR33] Gallese V (2003). The manifold nature of interpersonal relations: the quest for a common mechanism. Philos Trans R Soc Lond Ser B Biol Sci.

[CR34] Gallese V, Fadiga L, Fogassi L, Rizzolatti G (1996). Action recognition in the premotor cortex. Brain.

[CR35] Gazzola V, Aziz-Zadeh L, Keysers C (2006). Empathy and the somatotopic auditory mirror system in humans. Curr Biol.

[CR36] Geisser ME (2007). Surface electromyography and low back pain. Biofeedback.

[CR37] Geisser ME, Haig AJ, Theisen ME (2000). Activity avoidance and function in persons with chronic back pain. J Occup Rehabil.

[CR38] Hajcak G, Molnar C, George MS, Bolger K, Koola J, Nahas Z (2007). Emotion facilitates action: A transcranial magnetic stimulation study of motor cortex excitability during picture viewing. Psychophysiology.

[CR39] Hill AT, Fitzgibbon BM, Arnold SL, Rinehart NJ, Fitzgerald PB, Enticott PG (2013). Modulation of putative mirror neuron activity by both positively and negatively valenced affective stimuli: a TMS study. Behav Brain Res.

[CR40] Hodges PW (2001). Changes in motor planning of feedforward postural responses of the trunk muscles in low back pain. Exp Brain Res.

[CR41] Hodges PW, Moseley GL, Gabrielsson A, Gandevia SC (2003). Experimental muscle pain changes feedforward postural responses of the trunk muscles. Exp Brain Res.

[CR42] Hodges NJ, Williams AM, Hayes SJ, Breslin G (2007). What is modelled during observational learning?. J Sports Sci.

[CR43] Kalichman L, Hodges P, Li L, Guermazi A, Hunter DJ (2010). Changes in paraspinal muscles and their association with low back pain and spinal degeneration: CT study. Eur Spine J.

[CR44] Lapatki BG, Oostenveld R, Van Dijk JP, Jonas IE, Zwarts MJ, Stegeman DF (2010). Optimal placement of bipolar surface EMG electrodes in the face based on single motor unit analysis. Psychophysiology.

[CR45] Larsen JT, Norris CJ, Cacioppo JT (2003). Effects of positive and negative affect on electromyographic activity over zygomaticus major and corrugator supercilii. Psychophysiology.

[CR46] Lepage J, Tremblay S, Théoret H (2010). Early non-specific modulation of corticospinal excitability during action observation. Eur J Neurosci.

[CR47] MacDonald D, Moseley GL, Hodges PW (2010). People with recurrent low back pain respond differently to trunk loading despite remission from symptoms. Spine.

[CR48] Maher CG, Latimer J, Hodges PW, Refshauge KM, Moseley GL, Herbert RD (2005). The effect of motor control exercise versus placebo in patients with chronic low back pain [ACTRN012605000262606]. BMC Musculoskelet Disord.

[CR49] Mayer TG, Neblett R, Brede E, Gatchel RJ (2009). The quantified lumbar flexion-relaxation phenomenon is a useful measurement of improvement in a functional restoration program. Spine.

[CR50] McGorry RW, Lin J (2012). Flexion relaxation and its relation to pain and function over the duration of a back pain episode. PloS One.

[CR51] Ménoret M, Curie A, des Portes V, Nazir TA, Paulignan Y (2013). Simultaneous action execution and observation optimise grasping actions. Exp Brain Res.

[CR52] Morrison J (2004). Understanding others by understanding the self: neurobiological models of empathy and their relevance to personality disorders. Can Child Adolesc Psychiatry Rev.

[CR53] Morrison I, Poliakoff E, Gordon L, Downing P (2007). Response-specific effects of pain observation on motor behaviour. Cognition.

[CR54] Moseley GL, Hodges PW (2005). Are the changes in postural control associated with low back pain caused by pain interference?. Clin J Pain.

[CR55] Murata A, Wen W, Asama H (2016). The body and objects represented in the ventral stream of the parieto-premotor network. Neurosci Res.

[CR56] Ostelo RW, de Vet HC (2005). Clinically important outcomes in low back pain. Best Pract Res Clin Rheumatol.

[CR57] Sánchez-Zuriaga D, López-Pascual J, Garrido-Jaén D, García-Mas MA (2015). A comparison of lumbopelvic motion patterns and erector spinae behavior between asymptomatic subjects and patients with recurrent low back pain during pain-free periods. J Manipulative Physio Therapy.

[CR58] Schinkel-Ivy A, Nairn BC, Drake JD (2013). Evaluation of methods for the quantification of the flexion-relaxation phenomenon in the lumbar erector spinae muscles. J Manipulative Physio Therapy.

[CR59] Singer T, Seymour B, O’Doherty J, Kaube H, Dolan RJ, Frith CD (2004). Empathy for pain involves the affective but not sensory components of pain. Science.

[CR60] Smeets R, Köke A, Lin C, Ferreira M, Demoulin C (2011). Measures of function in low back pain/disorders: Low back pain rating scale (LBPRS), oswestry disability index (ODI), progressive isoinertial lifting evaluation (PILE), quebec back pain disability scale (QBPDS), and Roland-Morris disability questionnaire (RDQ). Arthritis Care Res.

[CR61] Soer R, Reneman MF, Speijer BL, Coppes MH, Vroomen PC (2012). Clinimetric properties of the EuroQol-5D in patients with chronic low back pain. Spine J.

[CR62] Tsao H, Druitt TR, Schollum TM, Hodges PW (2010). Motor training of the lumbar paraspinal muscles induces immediate changes in motor coordination in patients with recurrent low back pain. J Pain.

[CR63] Valeriani M, Betti V, Le Pera D, De Armas L, Miliucci R, Restuccia D (2008). Seeing the pain of others while being in pain: A laser-evoked potentials study. NeuroImage.

[CR64] van der Roer N, Ostelo RW, Bekkering GE, van Tulder MW, de Vet HC (2006). Minimal clinically important change for pain intensity, functional status, and general health status in patients with nonspecific low back pain. Spine.

[CR65] Villiger M, Chandrasekharan S, Welsh TN (2011). Activity of human motor system during action observation is modulated by object presence. Exp Brain Res.

[CR66] Vogt S, Thomaschke R (2007). From visuo-motor interactions to imitation learning: Behavioural and brain imaging studies. J Sports Sci.

[CR67] Watson P, Booker C, Main C, Chen A (1997). Surface electromyography in the identification of chronic low back pain patients: The development of the flexion relaxation ratio. Clin Biomech.

[CR68] de Williams AC (2002). Facial expression of pain: An evolutionary account. Behav Brain Sci.

[CR69] Wulf G, Mornell A (2008). Insights about practice from the perspective of motor learning: a review. Music Perform Res.

